# Clinical-Evolutionary Staging System of Primary Open-Angle Glaucoma Using Optical Coherence Tomography

**DOI:** 10.3390/jcm9051530

**Published:** 2020-05-19

**Authors:** Alfonso Parra-Blesa, Alfredo Sanchez-Alberca, Jose Javier Garcia-Medina

**Affiliations:** 1 Department of Ophthalmology, Policlínica Baza, 18800 Baza (Granada), Spain; 2 CEINDO, San Pablo CEU University, 28668 Madrid, Spain; 3 Department of Statistics and Applied Maths, San Pablo CEU University, 28668 Madrid, Spain; asalber@ceu.es; 4 Department of Ophthalmology, General University Hospital Morales Meseguer, 30100 Murcia, Spain; jj.garciamedina@um.es; 5 Department of Ophthalmology and Optometry, University of Murcia, 30100 Murcia, Spain

**Keywords:** primary open-angle glaucoma, retinal disease, spectral domain-optical coherence tomography, retinal imaging, evolutionary stages

## Abstract

Background: Primary open-angle glaucoma (POAG) is considered one of the main causes of blindness. Detection of POAG at early stages and classification into evolutionary stages is crucial to blindness prevention. Methods: 1001 patients were enrolled, of whom 766 were healthy subjects and 235 were ocular hypertensive or glaucomatous patients in different stages of the disease. Spectral domain optical coherence tomography (SD-OCT) was used to determine Bruch’s membrane opening-minimum rim width (BMO-MRW) and the thicknesses of peripapillary retinal nerve fibre layer (RNFL) rings with diameters of 3.0, 4.1 and 4.7 mm centred on the optic nerve. The BMO-MRW rim and RNFL rings were divided into seven sectors (G-T-TS-TI-N-NS-NI). The k-means algorithm and linear discriminant analysis were used to classify patients into disease stages. Results: We defined four glaucoma stages and provided a new model for classifying eyes into these stages, with an overall accuracy greater than 92% (88% when including healthy eyes). An online application was also implemented to predict the probability of glaucoma stage for any given eye. Conclusions: We propose a new objective algorithm for classifying POAG into clinical-evolutionary stages using SD-OCT.

## 1. Introduction

A correct classification is based on the data to which, of a set of categories, a new observation belongs. The main difficulty encountered in medical science is identifying a criterion that can frame all forms of a disease such as glaucoma in a coherent system to offer direct therapeutic suggestions [1]. The objective of this study was to identify an essential element that allows us to develop a clinical-evolutionary classification system for primary open-angle glaucoma (POAG).

Glaucoma is considered one of the main causes of blindness, and based on this relationship, the WHO specifically recommends early diagnosis and treatment for blindness prevention [2].

The currently accepted classification in Europe was proposed by the European Glaucoma Society in 2017 [1].

The visual field (VF) has provided information about the evolution of glaucoma and its diagnosis, but its subjective nature and late significance in relation to the onset of this disease, which requires loss of 40% of retinal ganglion cells, limit its use as an early diagnosis element [3].

A new technology called optical coherence tomography (OCT) emerged in 1991, based on the work of Huang and Fujimoto [4]. The non-invasive, objective and reproducible nature of OCT, coupled with its ability to quantify layers of the retina, help us to better monitor and diagnose glaucoma, especially in early stages [5,6,7,8,9,10,11,12].

This work aims to define a new clinical-evolutionary classification of POAG based on the k-means algorithm using objective RNFL and BMO-MRW data acquired from SD-OCT.

## 2. Materials and Methods

### 2.1. Study Design and Population

We planned an analytical, observational, prospective, cross-sectional and comparative study including healthy subjects, ocular hypertension patients and POAG patients. They were consecutively recruited at Policlínica Baza (Baza, Spain) and Clínica Vistacamacho (Almería, Spain) between May 2016 and June 2018.

The patients were informed about the nature of the study, agreed to participate, and] provided informed consent according to the data protection law currently in force. The study adhered to the conditions of the Declaration of Helsinki (sixth revision, 2008) and was approved by the ethics committees of both participating centres (Policlínica Baza, Baza, Spain and Clínica Vistacamacho, Almería, Spain).

The inclusion criteria were: (A) cognitive ability to accept and understand the proposed procedures; (B) a clinical record of intraocular pressure (IOP) < 23 with the absence of perimetric damage, normal optic disc appearance in the fundus examination and all sector values assessed by SD-OCT (BMO-MRW and peripapillary measurements) over the 5th percentile; (C) a clinical record of intraocular pressure IOP > 23 mmHg indicative of ocular hypertension (OH). In the absence of perimetric damage, normal optic disc appearance and all sector values assessed by SD-OCT over the 5th percentile; (D) POAG with historical clinical records of a IOP > 23 mmHg at least once, perimetric damage or glaucomatous optic disc appearance; such patients should have received anti-hypertensive treatment. (E) All the patients had to have a minimum of two clinical records: VF < 20% considering false-negative and false-positive responses; <20% fixation losses, as well as glaucomatous defects, according to the Hodapp classification. VF tests were performed using the standard Swedish 64 interactive threshold algorithm (SITA) target and the VF target strategy with a Humphrey II field analyser 65 (software version 4, programme 24-2, Goldmann objective size III, stimulus duration of 200 ms, model 66 HFA 740, Humphrey Instruments, Inc., Dublin, CA, USA); (F) Caucasian race; (G) open-angle verification by gonioscopy and the absence 68 of signs of pseudoexfoliation and pigmentary glaucoma.

The exclusion criteria: Ocular hypertensive patients or POAG patients were excluded from the study if they (A) did not agree to participate or did not meet the inclusion criteria, (B) had a direct family history of cognitive neurodegenerative diseases or exhibited suspicious signs, (C) had diseases affecting the retina and optic nerve before or at the time of recruitment, (D) had undergone intraocular surgeries in the previous 6 months, except for successful cataract removal or surgeries related to POAG, (E) exhibited non-transparent media at any level, (F) had severe systemic diseases, (G) had refractive errors above the spherical equivalent of three dioptres, (H) had SD-OCT tests with signal strengths below 20, or (I) were initially considered hypertensive and did not have high IOP values after 3 months of anti-hypertensive treatment and discontinuation of this treatment.

### 2.2. Ophthalmological Assessment

A complete ophthalmological examination was performed during the inclusion session, which included best-corrected visual acuity, cycloplegic refractions (Tropicamide 1%), visual acuity with best correction, measurement of central corneal thickness by ultrasound pachymetry, slit-lamp examination and gonioscopy, and a retinal and optic nerve funduscopy with a 78-dioptric hand lens.

### 2.3. Spectral Domain Optical Coherence Tomography

SD-OCT was performed on all patients on one side to avoid problems related to a correlation between the eyes of the same patient. OCT was performed with the Heidelberg Spectralis device (Heidelberg Engineering, GmbH, Heidelberg, Germany, Software Heidelberg Eye Explorer ver. 6.7c).

The glaucoma program provided by the manufacturer was used, which has a patented anatomical positioning system (APS) with a series of patterns for scanning of the optic nerve head, the RNFL and the macular ganglion cell layer. The program compares the eyes of patients with normalised baseline values for normal eyes. All the participants underwent an examination of the thicknesses of peripapillary RNFL rings with diameters of 3.0, 4.1, and 4.7 mm centred on the optic nerve and BMO-MRW.

No segmentations were performed manually for the RNFL values. Only small corrections were made by the same experienced operator (A.P.B.) to readjust Bruchs membrane endings during the BMO-MRW examination.

The absolute values of healthy, glaucomatous and hypertensive eyes prior to normalisation indicated by Heidelberg Engineering and obtained from the seven sectors of the BMO-MRW and the three peripapillary rings (G-TS-T-TI-NS-N-NI), were exported to a Microsoft Excel (version 2016; Microsoft Corporation, Redmon, WA, USA) table. These absolute values were adjusted for age and the area of the papillary cup, and were normalised following the Heidelberg Engineering indications using the following formula provided by Heidelberg Engineering:

(1) $$z_{i}=\frac{x_{i}-\bar{x}-b_{xe}\left(e_{i}-\bar{e}\right)-b_{xa}\left(a_{i}-\bar{a}\right)}{s_{x}}$$

where:

$x_{i}$ is the value of individual *i* in variable *x*.
$\bar{x}$ is the mean of variable *x*.
$s_{x}$ is the standard deviation of variable *x*.
$e_{i}$ is the age of individual *i*.
$\bar{e}$ is the mean age of healthy individuals at baseline.
$b_{xe}$ is the slope of the regression line of variable *x* on age.
$a_{i}$ is the area of the BMO-MRW of individual *i*.
$\bar{a}$ is the mean area of the BMO-MRW of healthy individuals at baseline.
$b_{xa}$ is the slope of the regression line of variable *x* over the area of the BMO-MRW.
$z_{i}$ is the standardized value of individual *i* in variable *x*.

### 2.4. Statistical Analysis

Data processing and analysis were performed with programmes R (version 3.5), RKWard (version 7.0) and rk.Teaching (package) (version 1.3) [13].

A descriptive summary of the main variables in the study was given by groups (healthy and glaucomatous eyes), including the mean and standard deviation.

The normality of RNFL variables was proven with the Kolmogorov–Smirnov test, and the homogeneity of variances was tested with Box’s M test using an α level of 0.05.

Pearson’s correlation coefficient was used for the correlation analysis of the variables. A strong correlation was considered for (r > 0.75) and a very strong correlation for (r > 0.9). The principal components analysis (PCA) was performed to reduce data dimensionality and to determine which combination of variables explained most of the variability of data. The PCA is a statistical method used to reduce data dimensionality by transforming the original variables (usually correlated) into a new set of linearly uncorrelated variables. The first principal component is a linear combination of variables that explain the widest variability in the data (maximum variance). The second principal component is a linear combination of variables with maximum variance, which is orthogonal to the first component [14].

The k-means method was followed to cluster eyes into glaucoma stages [14]. This algorithm groups eyes into *k* clusters or classes according to the Euclidean distance from the eye to the cluster centroids in the *n*-dimensional space of the variables. Every eye is assigned to the cluster with the closest centroid. The initial choice of cluster centroids was random. The number of clusters *k* was decided according to elbow criteria [15] by observing the reduction in intra-group variability when increasing the number of clusters. This criterion chooses the number of clusters where reduction in intra-group variability significantly decreases.

A linear discriminant analysis (LDA) [14] was used to classify eyes into the clusters, previously defined by the *k*-means method. LDA is a statistical method used to identify a set of discriminant functions, all of which are uncorrelated linear combinations of the variables, that maximizes the difference between classes and separates the classes the best [14]. The number of discriminant functions created is the number of classes minus one. The classification power of classifiers was assessed by cross-validation, and by computing sensitivity, specificity and the overall accuracy measures from the confusion matrix.

The data and all the results of this study are publicly available at https://github.com/asalber/glaucoma-staging and http://aprendeconalf.es/glaucoma-staging/.

## 3. Results

### 3.1. The Sample and Variables

For the study sample, 1001 individuals were selected (235 with glaucoma; 766 without glaucoma). To avoid a natural correlation between the eyes of the same individuals, only left eyes were considered. For each eye, the main variables measured through OCT were the thickness of the BMO-MRW at the temporal inferior (BMO-MRW.TI), temporal (BMO-MRW.T), temporal superior (BMO-MRW.TS), nasal superior (BMO-MRW.NS), nasal (BMO-MRW.N), nasal inferior (BMO-MRW.NI) and general (BMO-MRW.G) sectors, the thickness of the 3.5 ring of the RNFL at the temporal inferior (RNFL3.5.TI), temporal (RNFL3.5.T), temporal superior (RNFL3.5.TS), nasal superior (RNFL3.5.NS), nasal (RNFL3.5.N), nasal inferior (RNFL3.5.NI) and general (RNFL3.5.G) sectors, the thickness of the 4.1 ring of the RNFL at the temporal inferior (RNFL4.1.TI), temporal (RNFL4.1.T), temporal superior (RNFL4.1.TS), nasal superior (RNFL4.1.NS), nasal (RNFL4.1.N), nasal inferior (RNFL4.1.NI) and general (RNFL4.1.G) sectors, and the thickness of the 4.7 ring of the RNFL at the temporal inferior (RNFL4.7.TI), temporal (RNFL4.7.T), temporal superior (RNFL4.7.TS), nasal superior (RNFL4.7.NS), nasal (RNFL4.7.N), nasal inferior (RNFL4.7.NI) and general (RNFL4.7.G) sectors. The individuals’ ages and BMO-MRW areas were also documented for standardization. Table 1 shows the mean and the standard deviation of the variables herein considered.

### 3.2. Correlation Analysis

**Figure 1:**
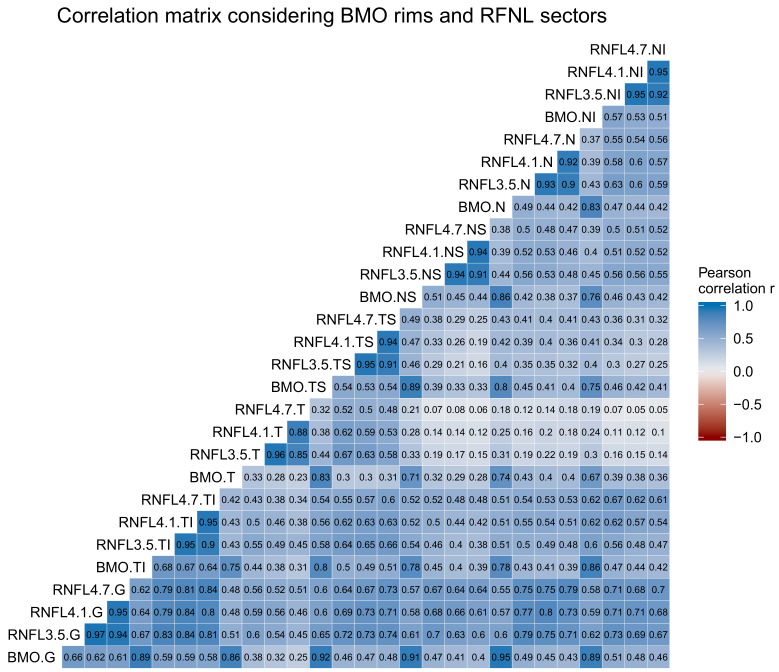
Correlation matrix map among the thickness of the BMO-MRW rims and RNFL sectors. Every cell represents the Pearson correlation coefficient r of the variables at the corresponding row and column. Dark blue represents a strong positive correlation, and light blue represents a weak positive correlation.

The correlation analysis (Figure 1) showed a clear correlation pattern by sectors. A very strong Pearson correlation (r>0.9) was observed among the thickness of the BMO-MRW and RNFL 3.5, 4.1 and 4.7 by sectors. A strong correlation (r>0.75) was also found among the sectors of the BMO-MRW, except for the BMO-MRW.G, which very strongly correlated with the other sectors. A very weak correlation (r<0.2) was found between the T sector and the N, NI and NS sectors.

### 3.3. Principal Component Analysis

As most of the variables were highly correlated, a PCA was performed to reduce the dimensionality and to determine which combination of variables explained most of the variability of the data.

**Figure 2:**
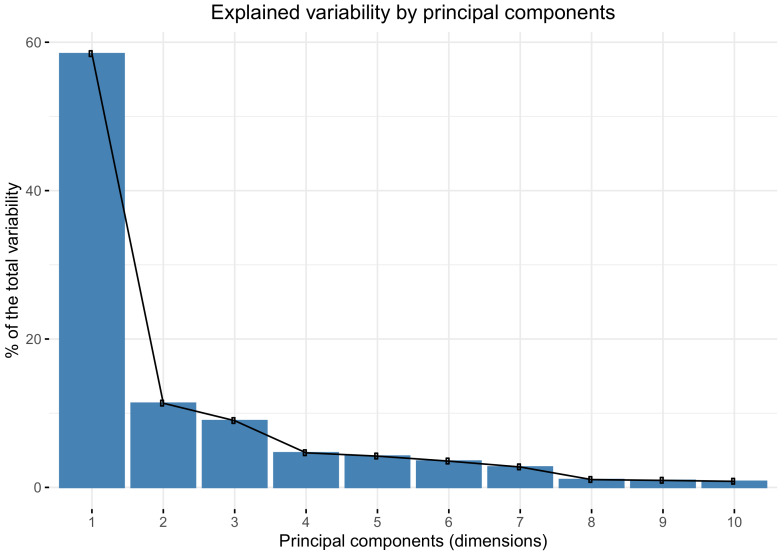
Percentage of the total variability explained by the main principal components.

Figure 2 shows that the first principal component explained almost 60% of the total variability of the data, while the second and third principal components explained only 11% and 9% of the total variability.

**Figure 3:**
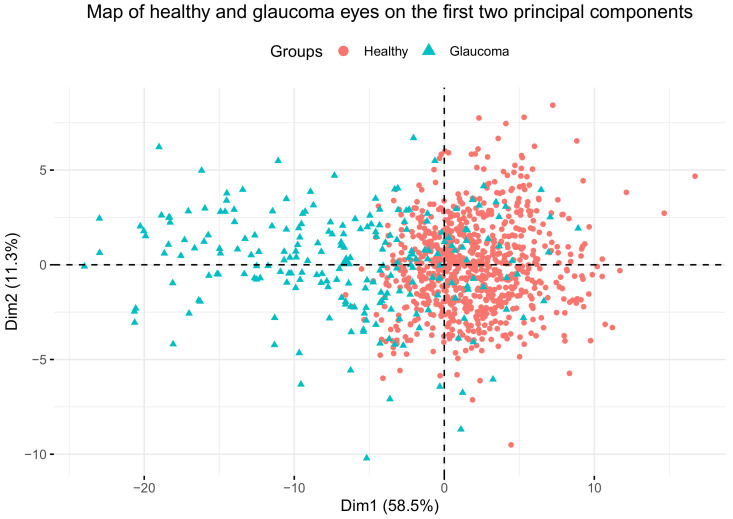
Map of healthy and glaucoma eyes on the first two principal components. The horizontal dimension (x-axis) corresponds to the first principal component, and the vertical dimension (y-axis) corresponds to the second principal component.

Plotting the subjects’ eyes on the first two principal components (Figure 3) revealed that healthy eyes could easily be discriminated from glaucoma eyes along the first principal component (horizontal dimension), with a small overlapping area, which was not possible at all along the second principal component (vertical dimension).

**Figure 4:**
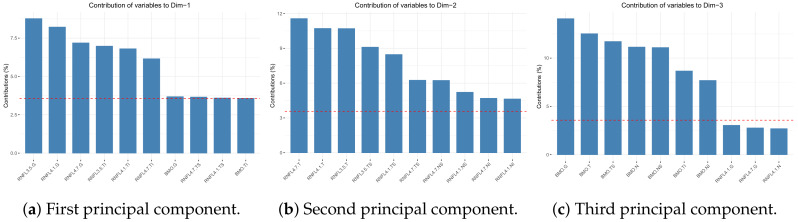
Contribution of variables to the main principal components.

According to Figure 4, the variables that more contributed to the first principal component were sectors G and TI of BMO-MRW and RNFL.

### 3.4. Cluster Analysis

**Figure 5:**
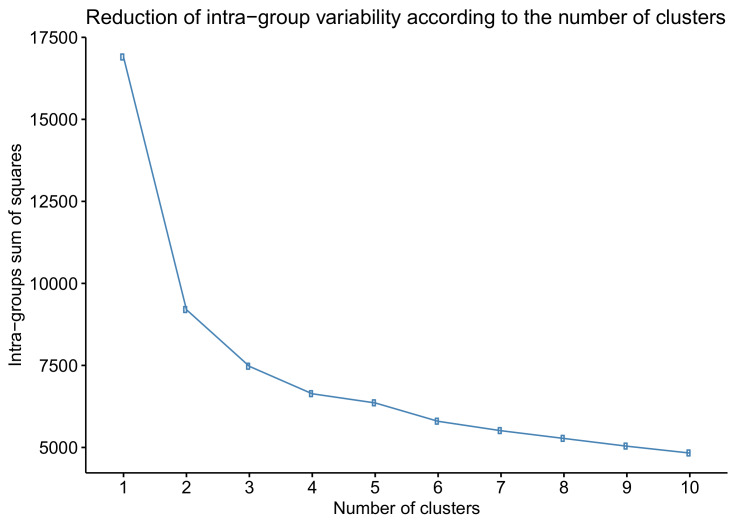
Reduction in the intra-group variability with an increasing number of clusters. According to the elbow criteria, the optimal number of clusters is 3 or 4.

To define the severity glaucoma stages, the k-means cluster algorithm was applied to the BMO-MRW and RNFL variables of eyes with glaucoma. Based on Figure 5, which shows the reduction in intra-group variability when increasing the number of clusters, we decided to create four clusters as a reduction in intra-group variability was not important for more than four groups (elbow criteria). We named these clusters I, II, III, and IV, which were in the order of increasing severity.

**Figure 6:**
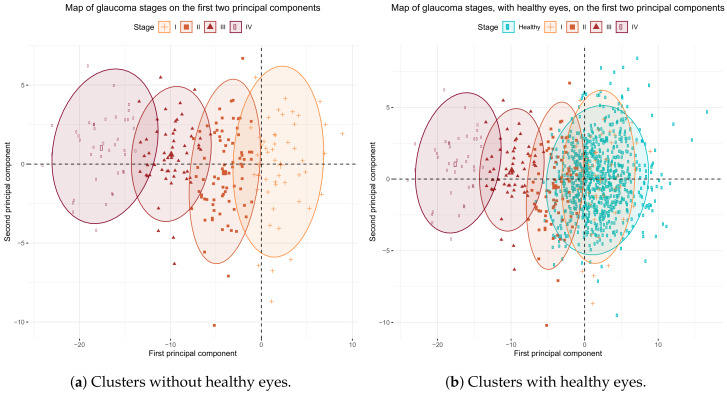
Clusters defined by the k-means method using all the BMO-MRW and RNFL sectors variables represented on the *x*-axis and *y*-axis corresponding to the first and second principal components, respectively.

Figure 6a shows the clusters on the axes corresponding to the first two principal components. As we can see in this chart, the four clusters can be separated almost perfectly along the *x*-axis of the first principal component.

**Figure 7:**
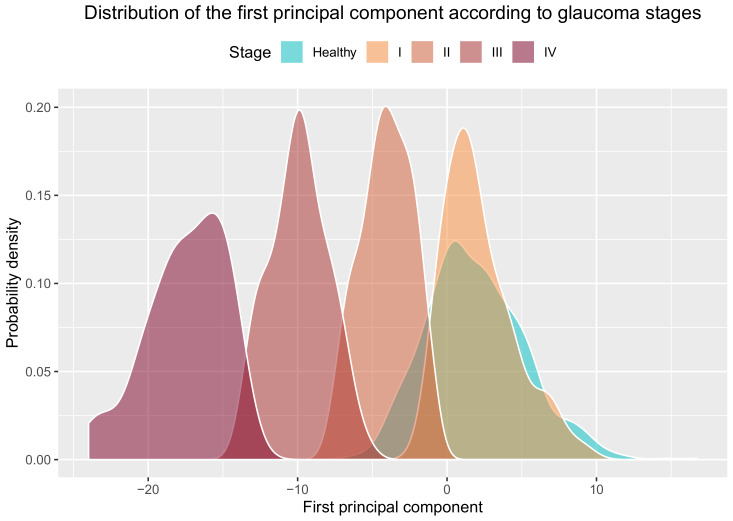
Distribution of the first principal component of the BMO-MRW and RNFL sectors variables by glaucoma stages and healthy eyes.

To determine whether the detected classes were related to glaucoma severity, that is, whether a gradient of severity existed from cluster I to cluster IV, we added healthy eyes to the previous plot. In Figure 6b, we see that healthy eyes were plotted to the right of the *x*-axis (corresponding to the first principal component), overlapping the eyes of cluster I and partially overlapping the eyes of cluster II. This finding is logical as cluster I contained eyes in the early glaucoma stages, which do not differ that much from healthy eyes. Clusters III and IV are located at the other end of the *x*-axis far from the healthy eyes, which correspond to eyes with severer glaucoma. We can see that cluster IV do not overlap with healthy eyes and the overlap of cluster III is minimal, which comes over more clearly in Figure 7 where the distribution of the first principal component for every glaucoma stage and healthy eyes is depicted.

### 3.5. Linear Discriminant Analysis

After defining clusters, we applied LDA to determine whether the four defined stages can be easily predicted. The LDA was applied to the principal components of the BMO-MRW rims and RNFL sectors as the original variables were highly correlated. Previously, we assessed the normality and homogeneity of variances of the principal components.

As shown in Table 2a and Table 3a, the four glaucoma classes could be predicted with very high sensitivity (>0.9) and specificity (>0.95), and 92.9% of the eyes were correctly classified.

We repeated the same analysis including healthy eyes. As expected, the classification power by LDA decreased, although this decrease was slight as we were still able to correctly classify 89% of eyes. Table 2b and Table 3b show the sensitivity, specificity, and balanced accuracy of the LDA classification. While specificity remained very high (except for healthy eyes), sensitivity decreased substantially for classes I and II because these two classes overlapped mostly healthy eyes. When two classes overlap, the LDA tends to classify an individual into the class with more instances, that is, the most likely class, which is the class of healthy eyes in this study.

### 3.6. Simplifying the Model

After defining the glaucoma classes and assessing whether these classes could be predicted fairly well, we examined whether these classes could be defined without using all the BMO-MRW rims and RNFL sectors, but using only the most important variables because we observed a strong correlation among most of them. So we repeated the LDA for different combinations of the BMO-MRW rims and RNFL sectors.

As the potential number of variable combinations was computationally impracticable, we focused on the variables that more contributed to the first principal component, which was the direction along which the separation of classes was clearer. These variables were sectors G and TI of BMO-MRW and RNFL, as shown in Figure 4a. Table 4 summarizes a comparison of the different assessed models.

The model with the highest overall accuracy was the model with rims G and TI of the BMO-MRW and all the RNFL sector variables (with and without including healthy eyes). However, the model that employed only the G and TI rims of BMO-MRW and the RNFL 3.5 sector achieved almost the same overall accuracy both with and without including healthy eyes. Regarding the sensitivity, specificity, and overall accuracy of this model, Table 5 and Table 6 shows that no big changes appeared in the model’s overall accuracy compared to Table 3 when all the variables were included.

**Figure 8:**
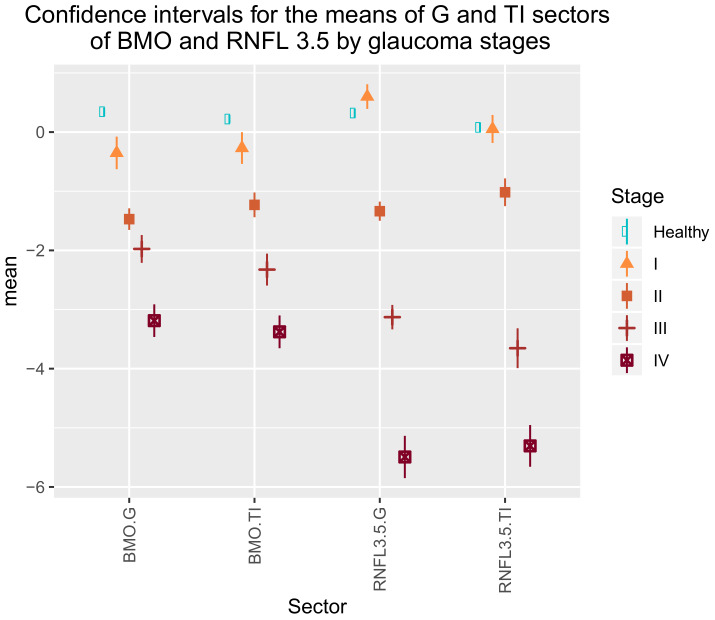
Distribution of the first principal component of the BMO-MRW and RNFL sector variables by glaucoma stages and healthy eyes.

Thus, we decided to use only the G and TI rims of the BMO-MRW and RNFL 3.5 sectors to classify eyes into glaucoma stages. Table 7 shows the coefficients of the model’s linear discriminant functions with these variables. Table 8 provides the main statistics for the distribution of these variables into glaucoma stages. Figure 8 graphically depicts the 95% confidence intervals for the means of these variables by glaucoma stages. As shown in this chart, classes are statistically well defined because for each G or TI sector variable, significant differences (α<0.05) were found among the means of all the stages, except for the means of the healthy and stage I eyes for the TI sector of the RNFL3.5. Another interesting observation was that the mean of the G sector of the RNFL3.5 for stage I glaucoma was higher than the mean of healthy eyes, while the mean for the G and TI rims of the BMO-MRW was lower. As stage I glaucoma overlapped mostly healthy eyes, this result reveals that the BMO-MRW is a better indicator when distinguishing stage I glaucoma eyes from healthy eyes.

**Figure 9:**
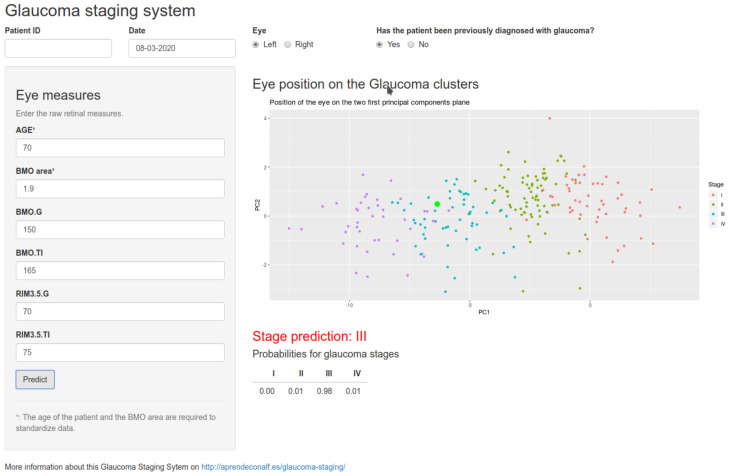
Classification of one eye using the glaucoma-staging app.

Finally, in order to render this staging system operational, we developed an online application (app) that implemented this classification model. Figure 9 shows a screenshot of the app. In this simple app, the user must provide a subject’s age and the BMO-MRW area, which are required for standardization, and the measurements for rims G and TI of the BMO-MRW and RNFL 3.5 sectors so that the app returns the glaucoma stage prediction for the eye and the probability for each stage. The app also plots the position of the eye on the two first principal components’ planes in relation to the clusters of the different stages. The app is currently available at https://asalberapps.shinyapps.io/Glaucoma-Staging-System/.

## 4. Discussion

Even after OCT emergence, one constant in the attempt made to classify POAG was the VF. Our study did not consider the VF to be a classification element given its subjective nature because of arbitrary limits of exclusion according to fixed losses, false-positives, cognitive impairment and difficult collaborations. VF-based classifications have been proposed, and many reviews of these classifications have been published [16,17,18]. A summary of all of these reviews may yield an inevitable conclusion. The presence of glaucomatous optic neuropathy increases with staging severity for all systems. However, different systems led to different severity stages [18].

Other proposals for the diagnosis and classification of POAG have recently been presented based on OCT angiography [19,20,21,22,23]. All of these proposals, and regardless of them being based on retrospective reviews of articles [23] or personal work, the potential of this technique is revealed, but they also introduce uncertainty due to inconsistent criteria in glaucoma diagnosis termes, especially the glaucoma classification. We believe that OCT angiography introduce elements such as anatomical anomalies, exudates, haemorrhages, and the distorting effect of the vessels on the nasal side of the papilla, which can lead to misinterpretation. Therefore, we considered the actual anatomical shape of the papilla limits, BMO-MRW [24], and the normalized values of the BMO-MRW and RNFL to be objective, accessible, and reproducible variables to propose a clinical-evolutionary glaucoma classification system.

The diagnostic accuracy of the sectoral and total analysis of RNFL and/or BMO-MRW has also been determined in glaucoma. Danthurebandara et al. [25] used an independent OCT normative database and classified eyes as glaucomatous if their BMO-MRW or RNFL values went below the normative limits of 1%, 5% or 10% of the normative database by using all the measurements (total analysis) or the sectoral means (sectoral analysis). They reported that at a normative limit of 1%, the sectoral analysis of BMO-MRW gave 87% sensitivity and 92% specificity, while the total analysis yielded 88% sensitivity at the same specificity (92%). For RNFLT, the sectoral analysis yielded 85% sensitivity and 95% specificity, while the total analysis gave 83% sensitivity at the same specificity (95%). The results for the 5% and 10% normative limits yielded lower specificity, but higher sensitivity. The authors concluded that both analyses, the sectoral and total, had similar diagnostic accuracies.

Fan et al. [26] compared the diagnostic ability between three-dimensional (3D) neuroretinal edge parameters (including BMO-MRW) and two-dimensional (2D) parameters (including RNFL thickness) using SD-OCT. They concluded that 3D parameters have the same or better diagnostic ability than 2D parameters. Among the three parameters of the 3D edges (Minimum Distance Band (MDB), BMO-MRW-MRW), no significant differences were found in diagnostic capacity (false detection rate >0.05 with 95% specificity).

Authors like Zheng et al. [27] have also worked with the inferotemporal and superotemporal sectors of the RNFL to improve both sensitivity and specificity in relation to the same sectors of the BMO-MRW with POAG perimetry and percentiles <5. Abnormal superotemporal and/or inferotemporal RNFL thicknesses achieved a higher sensitivity than abnormal superotemporal and/or inferotemporal BMO-MRWs in detecting mild glaucoma (mean VF DM: −3.32 ± 1.59 dB) (97.9% and 88.4%, respectively, *p* = 0.006), and glaucoma (mean VF DM: −9.36 ± 8.31 dB) (98.4% and 93.6%, respectively, *p* = 0.006), with the same specificity (96.1%). We belive that below the 5th percentile, the best definitions are of little significance when more specific sectors like G and TI are not studied.

In our study, we relied on artificial intelligence (AI) for its potential for detect, diagnose and classify glaucoma through the automated processing of large data sets and the early detection of new patterns of diseases [28]. We considered the parameter BMO-MRW to be important when defining the stages of the disease, as shown in Table 4. Valuing the diagnostic capacities of the parameters RNFL thickness, BMO-MRW yielded better diagnostic performance than the other parameters. At 95% specificity, the sensitivity of RNFLT, BMO-HRW, and BMO-MRW was 70%, 51%, and 81%, respectively. More studies have compared the diagnostic ability of BMO–MRW and peripapillary RNFL. Gardiner [29] found that peripapillary RNFL outperformed BMO–MRW during the follow-up assessment of glaucoma. In contrast, Chauhan et al. [30], Enders et al. [31] and Malik et al. [32] proved that BMO–MRW showed higher diagnostic ability compared to peripapillary RNFL. Bambo et al. [33] did not find any differences between diagnostic ability of peripapillary RNFL and BMO–MRW in glaucoma. In the present study, we integrated both strategies to achieve a better model.

In a recent study, Brusini [34] proposed a classification with similarities to our own classification, but also with some definitive differences. Brusini’s classification uses the upper and lower RNFL thickness values plotted on a Cartesian plane to classify glaucoma OCT results. The RNFL defects are classified into six stages of increasing severity and three classes of defect location (upper, lower, or diffuse defects). The diagram was created based on 302 OCT tests with 94 healthy controls and 284 patients affected by perimetric POAG.

Our study provides a more practical and simpler proposal and incorporates healthy, OHT and glaucomatous patients. We noted some substantial differences with he study by Brusini. Firstly, we included more variables that may suffer the effect of glaucoma (28 variables of BMO-MRW and RNFL sectors) than those Brusini included (only two RNFL sectors). Brusini did not consider BMO-MRW assessment. We corroborated the importance of BMO-MRW for determining glaucoma severity as two of its variables (BMO-MRW.G and BMO-MRW.TI) appear in the simplified classification model (Table 4).

Brusini considered the mean thicknesses of the upper and lower quadrants of RNFL, but ignored other variables because they are not reliable for determining structural damage, although no justification for excluding these variables was provided. We propose considering all the the RNFL and BMO-MRW sectors (NI, N, NS, TS, T, and TI), as well as a global measure (G) that averages 728 points in each rim. We selected those sectors with greater discriminatory power in the final classification model.

Brusini proposed six groups of increasing severity to classify POAG arbitraryly, but it does not obey objective or statistical criteria. Our work established four classes because this number is optimal for reducing intra-group variability according to the *k*-means algorithm. A bigger number of classes does not substantially reduce the total intra-group variability, as shown in Figure 5.

The groups that Brusini established are separated on the Cartesian plane in the direction of the two variables considered by taking intervals of equal length in the x- and y-axes. However, these axes do not correspond to the directions of the maximum variability of the data, which would be the directions of the principal components.

Our groups are very well separated in the direction of the first principal component, as shown in Figure 6. When using the *k*-means algorithm, the resulting groups were not same size, but emerge more naturally by agglutination around the centroids of each group. As for the validation of the classification model, Brusini’s only studied sensitivity (0.85–0.95) and specificity (0.92–0.98) to distinguish between patients with perimetric damage and healthy patients, but not for making the distinction between different stages. Our results, which include ocular hypertensive patients (without 315 perimetric damage), validate the system with similar balanced accuracies (Table 6) when healthy eyes are not included. When healthy eyes are included in the classification system, the sensitivity of stages I and II lowers. The reason for this is that the cluster of stage I, and partially the cluster of stage II, overlap the cluster of healthy eyes, as we can see in Figure 6b. Thus most of the eyes in stage I are wrongly classified as healthy, as are some of the eyes in stage II. However, the sensitivity and specificity of stages III and IV remain similar to those of Brusini’s study.

Our classification algorithm not only demonstrates its functionality within a group of known glaucomatous patients but can also be useful for the diagnosis of POAG in general population surveys because, although its performance in early glaucoma stages (I and II) shows low sensitivity and specificity, significant values are reached in more advanced stages (III and IV), thus enabling diagnosis with a mean global precision of 0.88. When we considered the robustness of our study due to the verification and evaluation of 1001 patients, 235 of whom had glaucoma at various disease stages, we can accept that only a single tomographic scan was considered for the study (the last scan performed). This test was considered the examination that would define the current process state.

This study has some limitations. As it is a cross-sectional study, its conclusions cannot be strictly considered to POAG progression in time and follow-up. More studies have to be done in this sense. Moreover, this work was performed with Caucasian participants, so its results should not be extended to other races. In adition, individuals with refractive error above the spherical equivalent of three dioptres were excluded from this study in order to avoid artifactual results of OCT. Hence this system cannot be applied to eyes with moderate to severe refractive errors, and external validity of the current study may be restricted according to ethnic or refractive factors. Finally, we only considered VF results as the inclusion/exclusion criteria in this work. Thus, we did not compare our proposed OCT clinical-evolutionary staging system with VF parameters. The reasons for this were the subjective nature and poor reliability of VF, as explained above. However, we consider that this issue should be addressed in further studies.

In conclusion, we present a new objective method to classify glaucoma patients according to both BMO-MRW and RNFL measurements that could be useful in clinical practice.

## Figures and Tables

**Table 1:** Descriptive summary of the main variables for healthy and glaucoma eyes.

Variable	Glaucoma	Mean	Std.Dev
Age	N	47.52	18.69
Age	Y	66.82	13.92
BMO.Area	N	1.98	0.40
BMO.Area	Y	1.99	0.43
BMO.G	N	350.02	61.44
BMO.G	Y	220.99	71.86
BMO.NS	N	402.48	82.48
BMO.NS	Y	242.72	86.30
BMO.N	N	381.96	72.74
BMO.N	Y	245.68	87.21
BMO.NI	N	421.77	74.73
BMO.NI	Y	272.73	97.87
BMO.TI	N	368.08	66.23
BMO.TI	Y	223.87	93.75
BMO.T	N	251.81	54.99
BMO.T	Y	167.87	54.27
BMO.TS	N	340.88	72.08
BMO.TS	Y	196.27	82.17
RNFL3.5.G	N	102.68	9.40
RNFL3.5.G	Y	78.33	19.22
RNFL3.5.NS	N	122.58	23.45
RNFL3.5.NS	Y	90.57	29.08
RNFL3.5.N	N	87.20	13.15
RNFL3.5.N	Y	68.08	19.45
RNFL3.5.NI	N	120.23	22.68
RNFL3.5.NI	Y	88.04	28.32
RNFL3.5.TI	N	154.01	19.29
RNFL3.5.TI	Y	109.00	38.58
RNFL3.5.T	N	70.65	10.49
RNFL3.5.T	Y	60.64	15.92
RNFL3.5.TS	N	129.45	21.00
RNFL3.5.TS	Y	93.89	31.43
RNFL4.1.G	N	87.95	8.41
RNFL4.1.G	Y	68.73	16.05
RNFL4.1.NS	N	99.04	20.86
RNFL4.1.NS	Y	75.30	24.70
RNFL4.1.N	N	72.08	11.39
RNFL4.1.N	Y	57.32	15.94
RNFL4.1.NI	N	95.64	19.05
RNFL4.1.NI	Y	72.32	21.83
RNFL4.1.TI	N	138.28	16.79
RNFL4.1.TI	Y	99.90	33.05
RNFL4.1.T	N	63.31	9.25
RNFL4.1.T	Y	56.21	14.40
RNFL4.1.TS	N	118.76	18.38
RNFL4.1.TS	Y	86.98	28.21
RNFL4.7.G	N	77.02	6.90
RNFL4.7.G	Y	61.43	14.25
RNFL4.7.NS	N	82.13	17.81
RNFL4.7.NS	Y	63.45	20.93
RNFL4.7.N	N	61.52	9.52
RNFL4.7.N	Y	50.38	14.07
RNFL4.7.NI	N	77.57	15.04
RNFL4.7.NI	Y	59.69	18.47
RNFL4.7.TI	N	124.82	15.11
RNFL4.7.TI	Y	91.84	31.15
RNFL4.7.T	N	58.02	8.40
RNFL4.7.T	Y	52.54	14.26
RNFL4.7.TS	N	109.47	15.52
RNFL4.7.TS	Y	81.43	25.25

**Table 2:** The confusion matrix of classification into glaucoma stages using LDA with all the variables.

(a) Classification without healthy eyes					
Predicted\Actual		I	II	III	IV
Stage I		47	2	0	0
Stage II		4	76	3	0
Stage III		0	0	50	4
Stage IV		0	0	3	37
(b) Classification with healthy eyes					
Predicted\Actual	Healthy	I	II	III	IV
Healthy	747	42	36	0	0
Stage I	9	9	2	1	0
Stage II	8	0	36	8	0
Stage III	1	0	4	45	10
Stage IV	0	0	0	2	31

**Table 3:** The sensitivity, specificity and balanced accuracy of classification into glaucoma stages using LDA with all the variables.

	Sensitivity	Specificity	Balanced Accuracy
(a) Classification without healthy eyes.			
Stage I	0.92	0.99	0.96
Stage II	0.97	0.95	0.96
Stage III	0.89	0.98	0.93
Stage IV	0.90	0.98	0.94
(b) Classification with healthy eyes.			
Stage Healthy	0.98	0.65	0.82
Stage I	0.18	0.99	0.58
Stage II	0.46	0.98	0.72
Stage III	0.80	0.98	0.89
Stage IV	0.76	1.00	0.88

**Table 4:** Overall accuracy for the prediction of glaucoma clusters defined with different combinations of variables by LDA.

Variables Used in the Model	Overall Accuracy without Healthy Eyes	Overall Accuracy with Healthy Eyes
All the variables	0.93	0.88
Rims G and TI of BMO and all the RNFL sectors	0.94	0.89
Rims G of BMO and all the RNFL sectors	0.88	0.88
Rims TI of BMO and all the RNFL sectors	0.71	0.84
**Rims G and TI of BMO and 3.5 RNFL sector**	**0.92**	**0.88**
Rims G and TI of BMO	0.59	0.84
Sectors G and TI of 3.5 RNFL	0.89	0.86
Rim G of BMO and 3.5 RNFL sector	0.89	0.88
Rim TI of BMO and 3.5 RNFL sector	0.70	0.84
Rim G of BMO	0.57	0.83
Sector G of 3.5 RNFL	0.87	0.86
Rim TI of BMO	0.60	0.82
Sector TI of 3.5 RNFL	0.64	0.82

**Table 5:** The confusion matrix of classification into glaucoma stages using LDA with the G and TI sectors of the BMO-MRW and RNFL 3.5.

(a) Classification without healthy eyes					
Predicted\Actual		I	II	III	IV
Stage I		45	0	0	0
Stage II		6	75	4	0
Stage III		0	3	50	3
Stage IV		0	0	2	38
(b) Classification with healthy eyes					
Predicted\Actual	Healthy	I	II	III	IV
Healthy	755	50	38	2	0
Stage I	2	0	0	0	0
Stage II	7	1	37	7	0
Stage III	1	0	3	46	4
Stage IV	0	0	0	1	37

**Table 6:** The sensitivity, specificity and balanced accuracy of classification into glaucoma stages using LDA with the G and TI sectors of the BMO-MRW and RNFL 3.5.

	Sensitivity	Specificity	Balanced Accuracy
(a) Classification without healthy eyes			
Stage I	0.88	1.00	0.94
Stage II	0.96	0.93	0.95
Stage III	0.89	0.96	0.93
Stage IV	0.93	0.99	0.96
(b) Classification with healthy eyes			
Stage Healthy	0.99	0.60	0.79
Stage I	0.00	1.00	0.50
Stage II	0.47	0.98	0.73
Stage III	0.82	0.99	0.91
Stage IV	0.90	1.00	0.95

**Table 7:** Linear discriminant coefficients of the models with the G and TI sectors of the BMO-MRW and RNFL 3.5.

(a) Model without healthy eyes				
	LD1	LD2	LD3	
BMO.G	−0.1881	0.7502	−0.91648	
BMO.TI	−0.0943	−0.2901	1.56602	
RNFL3.5.G	−0.8857	0.4810	0.08495	
RNFL3.5.TI	−0.4108	−0.6927	−0.52166	
(b) Model with healthy eyes				
	LD1	LD2	LD3	LD4
BMO.G	−0.2069	1.2241	−0.2007	−1.1987
BMO.TI	−0.1555	−0.4096	−0.2255	1.7378
RNFL3.5.G	−0.6034	−0.1298	1.0720	0.2322
RNFL3.5.TI	−0.2950	−0.4165	−0.8337	−0.6390

**Table 8:** The 95% confidence intervals for the means of the G and TI sectors of the BMO-MRW and RNFL 3.5 by glaucoma stages and healthy eyes.

Sector	Stage	n	Mean	sd	se	lower.ci	upper.ci
BMO.G	Healthy	765	0.34	1.04	0.04	0.27	0.41
BMO.G	I	51	−0.35	0.98	0.14	−0.63	−0.08
BMO.G	II	78	−1.47	0.81	0.09	−1.65	−1.29
BMO.G	III	56	−1.98	0.88	0.12	−2.21	−1.74
BMO.G	IV	41	−3.19	0.87	0.14	−3.46	−2.91
BMO.TI	Healthy	765	0.22	1.00	0.04	0.15	0.29
BMO.TI	I	51	−0.27	0.95	0.13	−0.54	−0.00
BMO.TI	II	78	−1.23	0.93	0.11	−1.44	−1.02
BMO.TI	III	56	−2.32	1.01	0.13	−2.59	−2.06
BMO.TI	IV	41	−3.38	0.88	0.14	−3.65	−3.10
RNFL3.5.G	Healthy	765	0.32	1.04	0.04	0.24	0.39
RNFL3.5.G	I	51	0.60	0.74	0.10	0.39	0.81
RNFL3.5.G	II	78	−1.34	0.72	0.08	−1.50	−1.18
RNFL3.5.G	III	56	−3.13	0.77	0.10	−3.34	−2.92
RNFL3.5.G	IV	41	−5.49	1.13	0.18	−5.85	−5.14
RNFL3.5.TI	Healthy	765	0.08	1.11	0.04	−0.00	0.15
RNFL3.5.TI	I	51	0.05	0.84	0.12	−0.18	0.29
RNFL3.5.TI	II	78	−1.02	1.04	0.12	−1.25	−0.78
RNFL3.5.TI	III	56	−3.65	1.26	0.17	−3.99	−3.32
RNFL3.5.TI	IV	41	−5.31	1.12	0.17	−5.66	−4.95
